# ﻿Descriptions of two new dark-body snake eels of the genus *Ophichthus* (Anguilliformes, Ophichthidae) from Taiwan

**DOI:** 10.3897/zookeys.1220.126337

**Published:** 2024-12-09

**Authors:** Yusuke Hibino, Hsuan-Ching Ho, Jian-Fu Huang

**Affiliations:** 1 Kitakyushu Museum of Natural History and Human History, Fukuoka 805-0071, Japan Kitakyushu Museum of Natural History and Human History Fukuoka Japan; 2 Department and Graduate Institute of Aquaculture, National Kaohsiung University of Science and Technology, Kaohsiung 811, Taiwan National Kaohsiung University of Science and Technology Kaohsiung Taiwan; 3 Australian Museum, Sydney 2010, Australia Australian Museum Sydney Australia; 4 Institute of Marine Biology, National Taiwan Ocean University, Keelung 202, Taiwan National Taiwan Ocean University Keelung Taiwan

**Keywords:** Biodiversity, Elopomorpha, ichthyology, taxonomy, Teleostei

## Abstract

Two new species of dark-body snake eels are described based on specimens collected from Taiwan. *Ophichthuskbalanensis***sp. nov.** has a long tail; dorsal-fin origin above posterior third of pectoral fin; tip of lower jaw anterior to anterior-nostril tube; two simple, pointed protrusions along upper lip; preoperculomandibular pores 6 or 7 + 3; teeth on jaws and vomer mostly uniserial, except for biserial on posterior portion of maxilla and anterior portion of symphysis of dentary; vertebral formula 12-55-153 and median fins with narrow dark margins, except the pale fin origins. *Ophichthusmultidentis***sp. nov.** has a dorsal-fin origin well behind gill opening; mainly 4 rows of teeth on jaws; no protrusions along upper lip; a smaller head; mean vertebral formula 24-64-163 and pale median fins. Based on some recent papers and our result, a revised key to species is herein provided.

## ﻿Introduction

The genus *Ophichthus* is the most speciose taxon in the snake eel family Ophichthidae, comprising more than 100 species worldwide, including undescribed ones (Hibino pers. data). The genus is also a major component of snake eels in Taiwan (more than one-third of all species; Ho et al. 2015, 2018, 2022). In the last decade, more than 20 new *Ophichthus* species have been described (e.g., McCosker et al. 2012; McCosker and Ho 2015; Hibino et al. 2019a, 2019b). All these species were collected from the Indo-western Pacific Ocean. More species are waiting to be investigated and named (Hibino and Ho pers. obs.).

McCosker and Ho (2015) recognized 18 *Ophichthus* species from Taiwan, which included two new species. In recent years, several authors continued to explore the eel diversity around Taiwan, which brought the total number of species in the genus to 22 (Hibino et al. 2019a, 2019b; Hibino 2019; Hibino and McCosker 2020; Ho et al. 2022).

During our survey, many different *Ophichthus* eels were collected from the fish landing ports, and two of them have been described recently (Hibino et al. 2019a; Ho et al. 2022). Three species in Taiwan, *Ophichthusaphotistos* McCosker & Chen, 2000, *Ophichthusmacrochir* (Bleeker, 1852), and *Ophichthusobtusus* McCosker, Ide & Endo, 2012 have distinctly uniformly dark body. A fourth species with dark body, *Ophichthuskusanagi* Hibino, McCosker & Tashiro, 2019, was described from Japan and subsequently reported from the Dongsha Islands (Ho et al. 2022).

In this study, several unidentified dark body specimens were found in the collections collected from around Taiwan in recent years. These specimens do not match any other nominal species, but they are two distinct new species. Herein, we provide descriptions of these two new species that possess distinct characters.

## ﻿Materials and methods

All methods for counts and measurements follow McCosker (2010). Measurements for total and tail lengths are taken by 300 or 600 mm rulers and others by digital calipers to the nearest 0.1 mm. Vertebral counts were made from radiograph films or digital radiograph photographs. Mean vertebral formula (**MVF**) is expressed as the average of predorsal, preanal, and total vertebrae, and vertebral formula (**VF**) is the solo number of each (Böhlke 1982). Terminology of head structures around lips (cf. protrusions) and head pore system follow Hibino et al. (2019b), and are abbreviated as **IO** (infraorbital pores), **SO** (supraorbital pores), **POM** (preoperculomandibular pores), and **ST** (supratemporal pores). Total and head lengths are abbreviated as **TL** and **HL**, respectively.

Specimens of types including new species were deposited at Pisces Collection of National Museum of Marine Biology & Aquarium, Pingtung, Taiwan (**NMMB-P**) and the National Taiwan Ocean University, Laboratory of Aquatic Ecology, Department of Aquaculture, Keelung, Taiwan (**TOU-AE**). Other materials were those deposited at the above and the National Zoological Museum of China, Institute of Zoology, Chinese Academy of Sciences, Beijing, China (**ASIZB**), Fisheries Research Laboratory, Mie University, Tsu, Mie, Japan (**FRLM**), Kagoshima University Museum, Kagoshima, Japan (**KAUM**), and National Museum of Nature & Science, Tsukuba, Ibaraki, Japan (**NSMT**). The data provided in key to species include our materials and those used by Hibino et al. (2019b), as well as comparative data provided in McCosker and Ho (2015), Hibino et al. (2019a), and Vo et al. (2019).

The information for the key and materials examined is from specimens mostly larger than 300 mm TL. We estimate some characters, such as tooth arrangement and shape of protrusions, which may have ontogenetical changes; however, the meristic and pore counts can be used for all sizes. The key to species and Table 2 are based on Hibino (2019), Hibino et al. (2019a, 2019b), Hibino and McCosker (2020), Ho et al. (2022), Lee and Asano (1997), McCosker and Ho (2015), comparative materials, and a future publication prepared by Quang Van Vo.

## ﻿Results

### ﻿Family Ophichthidae

**Genus *Ophichthus* Ahl, 1789**

#### ﻿ Ophichthus kbalanensis

Taxon classificationAnimaliaAnguilliformesOphichthidae

Hibino & Ho sp. nov.

7ADFD4E0-F42D-56CE-8DA4-AAFCB53675A2

https://zoobank.org/3A13F9F3-BD1E-4AF5-B1A3-4BBD7D604634

**Figure 1.**
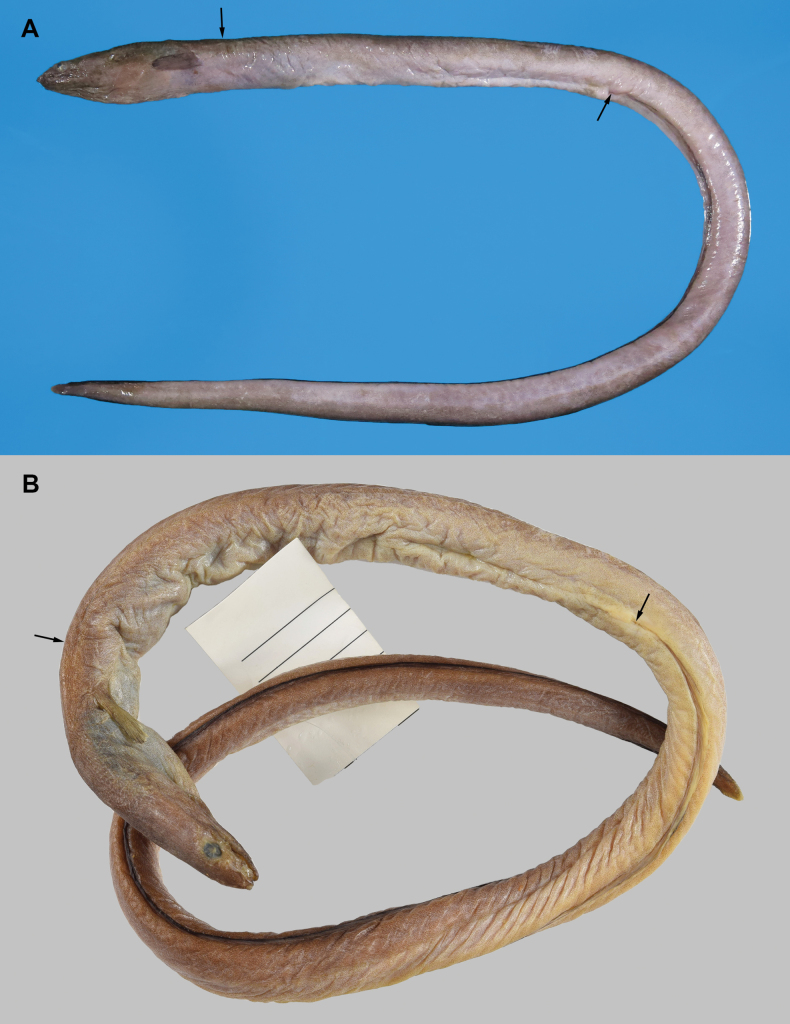
*Ophichthuskbalanensis* sp. nov., NMMB-P26381, holotype, 414 mm TL, Da-xi, Taiwan **A** fresh condition **B** preserved condition. Arrows indicate positions of dorsal-fin origin (lefts) and anus (rights).

**Figure 2.**
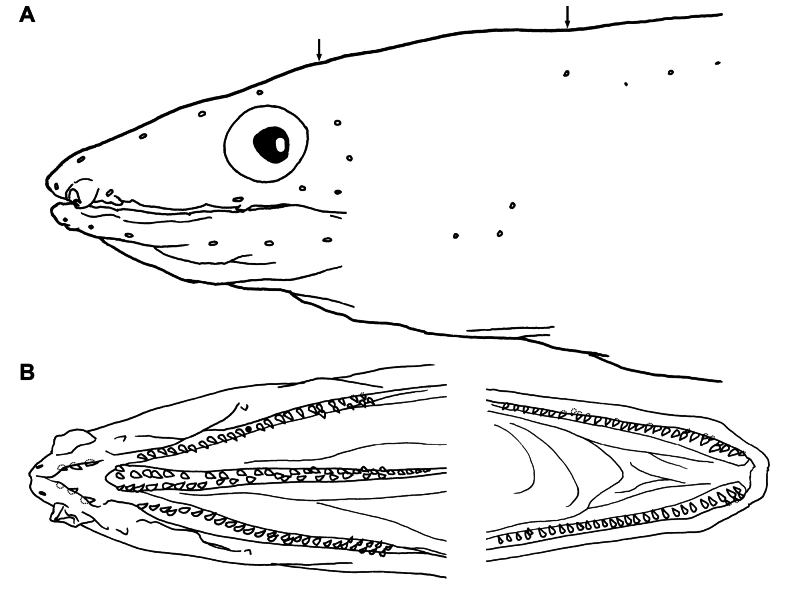
Line drawings of *O.kbalanensis* sp. nov., NMMB-P26381, holotype, 414 mm TL**A** sensory pores on head **B** teeth on upper (left) and lower jaws (right). Arrows indicate interorbital (left) and mid-temporal pores (right); black solid circle indicates holes of lost tooth; and broken lines indicate concealed teeth by fleshy lips.

Figs 1 , 2 ; Tables 1 , 2

##### Material examined.

***Holotype***: NMMB-P26381, 414 mm TL, ca 24°54.0'N, 121°56.0'E, Da-xi, Yilan, northeastern Taiwan, northwestern Pacific Ocean, 1 Jul. 2017.

##### Diagnosis.

A relatively short *Ophichthus* with the following combination of characters: head 10.3% TL; tail 62.7% TL; dorsal-fin origin above about middle of pectoral fin; tip of lower jaw anterior to anterior-nostril base; two simple, distally pointed protrusions along upper lip; SO 1 + 4; POM 6 or 7 + 3; teeth on jaws and vomer mostly uniserial but posterior ends of maxilla and anterior end of symphysis biserial; body dark; median fins with narrow dark margins, except the pale fin origins; 14 predorsal and 53 preanal lateral-line pores; VF 12-55-153.

##### Description.

Counts and measurements are mostly shown in Tables 1, 2.

**Table 1.** Counts and measurements of two new *Ophichthus* from Taiwan.

	*O.kbalanensis* sp. nov.	*O.multidentis* sp. nov.				
	Holotype	Holotype	Paratypes			
	NMMB-P26381	NMMB-P36205	TOU-AE 7802	TOU-AE 8998	TOU-AE 8999	TOU-AE 9294
Total length (mm)	414	433	519	554	597	696
As % of TL						
Head length (HL)	10.3	8.1	8.0	8.2	8.8	8.5
Preanal length	37.3	41.2	41.5	43.2	43.2	42.9
Tail length	62.7	58.8	58.5	56.8	56.8	57.1
Predorsal length	12.6	16.5	15.1	16.1	17.4	18.0
Body depth at gill opening	3.1	2.2	2.7	2.4	2.7	3.3
Body width at gill opening	2.0	1.6	2.3	1.7	1.9	2.7
Body depth at mid-anus	2.5	2.1	2.9	2.3	3.0	2.9
Body width at mid-anus	2.4	2.1	2.8	2.4	2.6	3.0
As % of HL						
Snout length	16.4	20.6	20.1	21.2	18.8	20.9
Eye diameter	6.8	8.6	9.2	8.3	8.4	9.1
Upper-jaw length	27.9	28.6	28.1	29.5	29.8	28.3
Gill-opening length	9.2	11.1	8.2	9.4	9.7	11.3
Interorbital width	9.9	10.0	10.2	12.7	12.9	16.5
Isthmus width	15.0	16.0	22.8	13.8	16.9	28.2
Pectoral-fin length	27.9	26.0	24.2	29.8	29.5	24.6
Pectoral-fin base	12.0	11.7	9.7	9.6	10.8	10.1
Counts						
Predorsal vertebrae	13	24	23	23	25	26
Preanal vertebrae	55	62	64	66	65	65
Total vertebrae	153	163	162	163	164	162

**Table 2.** Selected characters of *Ophichthus* species reported from Taiwan, except patterned species.

	SO	POM	Protrusion number	PALL	PDV	PAV	TV	TYPE V	Protrusion shape
*O.aphotistos*	1+4	6+2	0	59–61	16–19	57–61	157–162	18/59/161	Absent
*O.apicalis*	1+4	5 or 6+3	2	NO DATA	12–14	50–53	138–141	NO TYPE	Small, thorn-shape
*O.asakusae*	1+4	7-10+3	0 or 1	51–58	10–12	49–57	123–132	11/54/128	Robust hump shape, weak in smaller specimens
*O.bicolor*	1+4	6+2	0	63–67	15–23	61–66	155–163	19/65/160	Absent
*O.kbalanensis* sp. nov.	1+4	6 or 7+3	2	53	12	55	153	12/55/153	Simple thorn-shape
*O.kusanagi*	1+4	6+2	0	61–65	17–22	59–62	158–163	18/61/161	Absent
*O.machidai*	1+4	5 or 6+2 or 3	2	51–59	11–16	52–59	150–161	16/58/158	Simple thorn-shape
*O.macrochir*	1+4	4-6+2	2	68–73	11–12	67–71	207–221	11/70/221	Simple thorn-shape
*O.megalops*	1+4	6+3	0	59–64	28–35	59–63	157–168	29/60/160	Absent
*O.multidentis* sp. nov.	1+3	5+2	0	63	24	62	163	24/62/163	Absent
*O.obtusus*	1+4	4 or 5+3	2	57	11–19	52–57	148–159	12/57/151	Stout, with wrinkles in larger specimens
*O.pratasensis*	1+4	6+2	0	59–60	20	59	177	20/59/177	Absent
*O.rotundus*	1+3	5+2	2	65–66	14?	64?	178–184	14/64/182	Short, simple thorn-shape
*O.sangjuensis*	1+4	5 or 6+3	2	53	13–14	48–52	143–153	13/50/153	Simple thorn-shape
*O.semilunatus*	1+3	7+2	0	65	29	64	176	29/64/176	Absent
*O.shaoi*	1+4	6 or 7+3	1	69–72	10–13	68–72	155–168	12/68/155	Small, thorn-shape
*O.urolophus*	1+3 or 4	5-8+3	1	51–58	13–18	51–56	134–140	16/54/136	Robust hump shape, weak in smaller specimens
*O.zophistius*	1+4	5 or 6+3	2	59–64	11–13	61–63	177–184	12/62/181	Simple thorn-shape

Body elongate, but relatively short, subcylindrical, its depth at gill opening 11.9 in head and trunk, 31.8 in TL (Fig. 1); tail more compressed, tapering slowly towards tip of tail, its length 1.6 in TL. Skin of body wrinkled; relatively strong wrinkles on snout, with numerous fine longitudinal wrinkles on remaining head and body.

Head moderate, 3.6 in head and trunk and 9.7 in TL; dorsal contour of head weakly curved above eye, occipital weakly convex; branchial basket slightly swollen, its maximum depth 2.9 in head. Snout tip relatively blunt and robust, moderate in length, 6.1 in HL and 0.4 in eye. Anterior nostril a simple tube opening anteroventrally; posterior nostril a hole at inner margin of upper lip, completely covered by a wide dermal flap. Eye relatively small, 2.4 in snout length. Mouth subterminal, tip of lower jaw anterior to anterior base of anterior nostril tube. Rictus well behind posterior margin of eye. Lips smooth without small papillae; two low, small, simple, thorn-shaped protrusions, their tips pointed. Interorbital region smooth, transverse contour rounded, convex. Gill openings located ventrolaterally, upper ends slightly above middle of pectoral fin.

Sensory pores on head (Fig. 2A) developed but very small, not obvious; SO 1 + 4, first one (ethmoid) on ventral surface of snout; IO 3 + 3, the first behind anterior nostril base, 2 below eye, and 3 arranged in a vertical row behind eye; POM 6 (left) or 7 (right) + 3, the 6 (or seventh on right side) below rictus; ST 3, single pore on mid-temporal; and single interorbital pore. Lateral-line nearly complete, end anterior to about 1/2 HL before tail tip; canal on branchial basket slightly arched, 10 on branchial basket before gill opening, 14 anterior to origin of dorsal fin, 53 anterior to anus, and total 138.

Teeth moderate, conical, pointed (Fig. 2B); teeth on maxilla mostly uniserial, ending with an additional row consisting of 5 teeth on inner side; dentary mostly uniserial but an additional row of small teeth in outer region of symphysis anteriorly; vomerine biserial anteriorly and medially, uniserial at posterior end; 6 large teeth on intermaxillary, arranged as chevron-shape.

Dorsal and anal fins low, anal fin slightly higher than dorsal fin; both ending slightly anterior to tail tip. Dorsal-fin origin over about posterior third of pectoral fin. Pectoral fin tip weakly pointed, not lanceolate (somewhat damaged by trawl operation). Caudal fin absent, rear end of tail tip pointed.

##### Coloration.

Freshly caught specimen has a somewhat purplish body, darker dorsally and paler ventrally; pectoral fin dark brown and anal fin with dark brown to black margin; tail tip relatively pale (Fig. 1A). Preserved condition in 50% isopropanol ethanol (Fig. 1B): head and body dark brown, abdomen slightly paler, densely covered with melanophores; branchial basket darker and blueish. Anterior nostril tube similar to body color; sensory pores not prominently margined. Mouth cavity dusky brown. Membrane of gill opening paler than body. Anterior portion of dorsal fin creamy white, gradually becoming dark brown similar in color to dorsal surface; narrow black margin along entire dorsal fin. Anterior portion of anal fin creamy white, gradually becoming dark brown on anterior one-third of its length, with black margin becoming broader posteriorly. Pectoral fin yellowish brown, gradually darkening posteriorly.

##### Etymology.

The specific name is derived from the type locality “Kbalan”, an old name of Yilan region (Kat-má-lán in Taiwanese or Cabaran in Spanish) dated back to 1300–800 years ago. Kbalan means “people who live in the plain” in the Taiwanese aboriginal race Kebalan. The earliest record of Kbalan occurred in the occupation of the Spanish (~1632) which was replaced by the Dutch East India Company in 1642.

##### Comparison.

The first distinctive character found in *Ophichthuskbalanensis* sp. nov. is the unique tooth arrangement. In most species of *Ophichthus* we examined, the tooth rows on jaws maintained the same number or reduced to fewer row(s) posteriorly. However, in the new species, there is a short additional row of teeth on posterior portion of upper jaw.

Secondly, the tip of lower jaw extends beyond anterior margin of base of anterior nostril tube is also quite distinct among *Ophichthus* species (Hibino pers. obs.). *Ophichthusishiyamorum* McCosker, 2010 shares this character with *O.kbalanensis*, as well as the dorsal-fin origin above middle of the pectoral fin, and similar body coloration. However, *O.kbalanensis* sp. nov. differs from *O.ishiyamorum* in having a smaller head (10.3% TL vs 14–15% TL), more vertebrae (153 vs 130–132), maxillary teeth mostly uniserial but ending in biserial (vs mostly uniserial and biserial anteriorly), and median fins with dark margined (vs pale) (McCosker 2010).

The tip of lower jaw is also before the anterior nostril tube in *Ophichthusalleni* McCosker, 2010, several specimens of *Ophichthusasakusae* Jordan & Snyder, 1901 and *Ophichthusurolophus* (Temminck & Schlegel, 1846); however, they have only one or no protrusions on upper lip, much fewer total vertebrae (131–133 in *O.alleni*, 126–132 in *O.asakusae* and 134–139 in *O.urolophus*), and a bicolored body with a mostly pale ventral surface (McCosker 2010; Hibino et al. 2019b; this study).

In Taiwan, *O.kbalanensis* sp. nov. is also similar to *O.obtusus* in the uniformly black body and vertebral count, but it can be distinguished by the different tooth arrangement on the jaws, more mandibular pores (6 or 7 vs 4 or 5), position of the end of the rictus (behind posterior margin of eye vs not behind), and two small, simple, thorn-like labial protrusions on the upper lip (vs at least anterior one fat, cauliflower-shaped protrusion with weak wrinkles) (McCosker et al. 2012; Chiu et al. 2013; this study).

#### ﻿ Ophichthus multidentis

Taxon classificationAnimaliaAnguilliformesOphichthidae

Hibino, Ho & Huang sp. nov.

436BC030-8758-5134-AC4B-88D34B1B346A

https://zoobank.org/56CD9529-C140-4DCE-8691-59492233FB42

**Figure 3.**
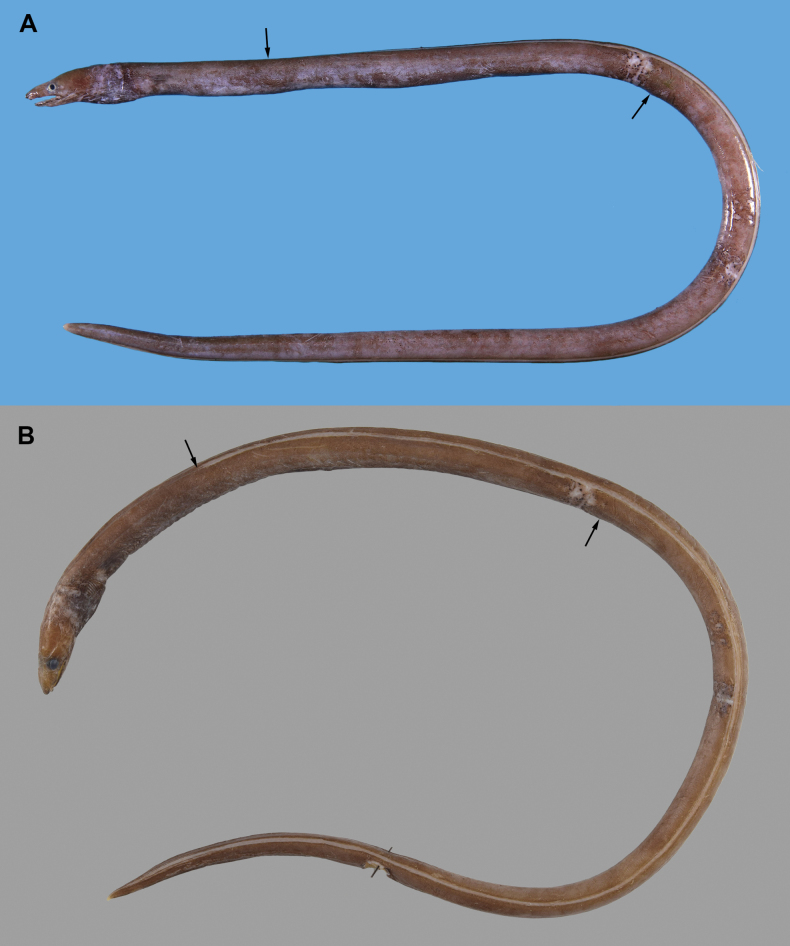
*Ophichthusmultidentis* sp. nov., NMMB-P36205, holotype, 433 mm TL, Ke-tzu-liao, Kaohsiung, Taiwan **A** fresh condition **B** preserved condition. Arrows indicate positions of dorsal-fin origin (lefts) and anus (rights).

**Figure 4.**
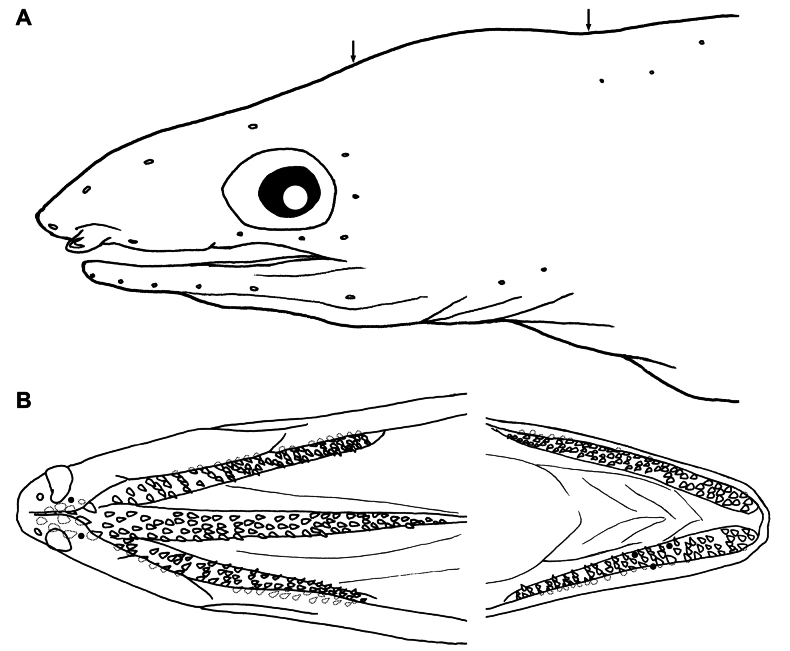
Line drawings of *O.multidentis* sp. nov., NMMB-P36205, holotype, 433 mm TL**A** sensory pores on head **B** teeth on upper (left) and lower jaws (right). Arrows indicate interorbital (left) and mid-temporal pores (right); black solid circles indicate holes of lost tooth; and broken lines indicate concealed teeth by fleshy lips.

Figs 3 , 4 ; Tables 1 , 2

##### Material examined.

***Holotype***: NMMB-P36205, 433 mm TL, ca 22°42.5'N, 120°10.8'E, off Ke-tzu-liao, Kaohsiung, southwestern Taiwan, northern South China Sea, 4 Sep. 2019, collected by H.-C, Ho. ***Paratypes***: Four specimens, all collected from Daxi fish landing port, 24°56.5'N, 121°54.0'E, northeastern Taiwan, southern East China Sea, collected by J.-F. Huang: TOU-AE 7802, 519 mm TL, 25 July 2020; TOU-AE 8998, 554 mm TL, TOU-AE 8999, 597 mm TL, 28 Nov. 2022; TOU-AE 9294, 696 mm TL, 9 Jan. 2023.

##### Diagnosis.

An elongate *Ophichthus* with the following combination of characters: head 8.0–8.8% TL; tail 56.8–58.8% TL; dorsal-fin origin behind pectoral-fin tip by 3.0 times the pectoral fin length; no protrusions along upper lip; SO 1 + 3 ; POM 5 or 6 + 2; teeth on maxilla in 4 irregular rows or 5 rows anteriorly and 4 rows posteriorly, teeth on vomer in up to 4 rows, teeth on dentary in 4 rows anteriorly, 3 rows posteriorly; body uniformly dark with creamy white median fins; 22–26 predorsal and 63–65 preanal lateral-line pores; total vertebrae 162–164, MVF 24-64-163.

##### Description.

Counts and measurements are mostly shown in Tables 1, 2.

Body elongate, slender (Fig. 3), cylindrical, its depth at gill opening 13.1–18.4 (18.4 in holotype) in head and trunk, 30.5–44.7 (44.7 in holotype) in TL; tail weakly compressed, tapering slowly toward tip, its length 1.7–1.8 (1.7 in holotype) in TL; skin of body nearly smooth, with numerous extremely small longitudinal wrinkles ventrolaterally.

Head small, 4.9–5.2 (5.1 in holotype) in head and trunk and 11.3–12.6 (12.4 in holotype) in TL; dorsal contour of head relatively linear above eye, occipital weakly convex; branchial basket moderately swollen, maximum depth 3.2 in head. Snout relatively acute but bulbous, relatively long, 4.7–5.3 (4.9 in holotype) in HL; a dermal groove ventrally on snout. Anterior nostril tubular, towards anteriorly; opening with moderately expanded flap anteriorly; posterior nostril a hole at inner margin of upper lip, completely covered by a wide but low dermal flap. Eye moderate in size, 1.7–2.6 (2.4 in holotype) times in snout length. Mouth inferior, tip of lower jaw below middle of anterior nostril tube base; rictus slightly behind posterior margin of eye. Lips without any sensory papillae and protrusions; inside along base on anterior nostril tube with several small low hump arranged as a row. Interorbital region smooth, gently convex. Gill openings located ventrolaterally, upper ends slightly below insertion of pectoral fin.

Sensory pores on head (Fig. 4A) developed but very small and not obvious; SO 1 + 3, first (ethmoid pore) on underside of snout tip and 3 along dorsal surface of snout, the last above upper margin of eye; IO 3 + 3, 1 pore behind base of anterior nostril, 2 below eye, and 3 arranged in a vertical row behind eye; POM 5 or 6 + 2 (6 in holotype), last pore behind rictus; ST 3, 1 on mid-temporal; single interorbital pore. Lateral line nearly complete, ending by about 1 HL before tail tip; canal on branchial basket weakly arched, 8 on branchial basket before gill opening, 22–26 anterior to origin of dorsal fin, 63–65 anterior to anus, and total 145–148 (8, 24, 63 and 145 in holotype).

Teeth numerous, conical, pointed but shape and size variable (Fig. 4B); multiserial teeth on maxilla and dentary, outermost rows slightly larger and more robust than others, innermost teeth slender and more recurved posteriorly. Those on maxilla in 4 irregular rows in holotype, but in larger specimens (TOU-AE 8999 and 9294) 5 rows anteriorly and 4 rows posteriorly; on dentary arranged in 4 rows anteriorly and 3 rows posteriorly; on vomer moderate in size, becoming smaller posteriorly, 4 rows maximum and decreasing to single row posteriorly; 6 or 7 (6 in holotype) large and robust, close-set teeth on intermaxillary, mostly concealed by lips.

Dorsal and anal fins low; ending slightly before tail tip; dorsal-fin origin well behind pectoral-fin tip by 2.3–3.5 (2.8 in holotype) times the pectoral-fin length; pectoral-fin tip pointed but not lanceolate; caudal fin absent.

##### Coloration.

Freshly caught specimen has a uniformly purplish to blackish brown body, pectoral fin dark gray, and dorsal and anal fins pale; tail tip pale (Fig. 4A). Preserved condition in 50% isopropanol ethanol (Fig. 3B): head and body dark brown except pale anus and tail tip; lips relatively darker than other skin; branchial basket and chest darker; anterior nostril tube same as body color; sensory pores not prominently margined; mouth cavity various, completely creamy white or dusky anteriorly, completely dusky on inside of lower jaw in three specimens; membrane of gill opening paler than body; head partly faded with some pale patches possibly imprinted by fishing net; dorsal and anal fins creamy white without melanophores; pectoral fin dark brown.

##### Etymology.

The specific name is derived from the Latin *multi* (many) and *dentes* (teeth), referring to the diagnostic character of four tooth rows on jaws.

##### Comparison.

The tooth pattern of *Ophichthusmultidentis* sp. nov. is unique among the congeners. It is the only member of *Ophichthus* that possesses up to 5 rows of small teeth on jaws and vomer in the northwestern Pacific region.

*Ophichthusmultidentis* sp. nov. is similar to a number of species that have the dorsal-fin origin situated behind the head by more than twice the pectoral-fin length (or predorsal length more than 1.5 times head length) and no blackened anal-fin base in advance of tail tip (Vo and Ho 2021: table 1; Ho et al. 2022). *Ophichthusmultidentis* sp. nov. is most similar to *Ophichthuslongicorpus* Vo & Ho, 2021 in having a uniformly dark body, dorsal-fin origin 3 times the pectoral-fin length behind the pectoral fin tip (cf. 3.4–5.7 times in *O.longicorpus*), but differs in having more tooth rows on jaws and vomer (at least 4 irregular rows on maxilla and vomer vs mainly 2 rows on jaws and 2 or 3 rows anteriorly and uniserial posteriorly on vomer), a longer tail (56.8–58.8% TL vs 50.0–52.9% TL), and different vertebral formula (MVF 24-64-163 vs 27-68-159).

*Ophichthusmultidentis* sp. nov. is also similar to *O.aphotistos* and *O.kusanagi* in having a uniformly colored body, similar vertebral counts and proportions of head length, tail length, and body depth. It differs from these species in having more predorsal vertebrae (23–26 vs 16–20 in *O.aphotistos* and 17–22 in *O.kusanagi*), more numerous and smaller teeth on maxilla arranged irregularly in up to 5 rows (vs relatively few and large teeth, arranged in biserial or mostly biserial anteriorly) and fewer mandibular pores (5 or 6 vs 6) (Hibino et al. 2016; Hibino et al. 2019b; this study). We do not have the sufficient information of the molecular evidence of the new species with other congeners, while the partial sequence of *O.multidens* sp. nov. differs from *O.aphotistos* (S. Endo pers. comm.).

### ﻿Key to species of *Ophichthus* from Taiwan, including the Dongsha Islands

**Table:** 

1	Body coloration markedly spotted or with distinct blotches or saddles	**2**
–	Body coloration uniform or darker dorsally, without distinct spots, blotches or saddles (rarely obscure distorted bars present dorsally)	**6**
2	A dark brown or black saddle on posterior half of head; body brown, without spots or prominent saddles but with or without irregular markings present	**3**
–	Head without a broad dark dorsal saddle; distinct spots, blotches, or saddles on body	**4**
3	Body with irregular markings dorsally; median fins without broad white margin	***O.lithinus* (Jordan & Richardson, 1908)**
–	Body pale, without markings; median fins dark brown with broad white margin	***O.cephalozona* Bleeker, 1864**
4	Body with 18–27 distinct black saddles, head with golden to brownish (in life) dark-margined marbling and spots; total vertebrae 156–164	***O.bonaparti* (Kaup, 1856)**
–	Head and body without bars, with spots only; total vertebrae 141–155	**5**
5	Head and body overlain with numerous ocellated spots, those on body in 3 regular alternating rows, the spots separated by pale interspaces; total vertebrae141–148	***O.polyophthalmus* Bleeker, 1864**
–	Head and body overlain with numerous brown spots, those on body in 2–4 irregular rows, the spots about equal in size to their interspaces; total vertebrae 151–155	***O.erabo* (Jordan & Snyder, 1901)**
6	Body extremely slender, depth at gill openings 1.6–2.3% TL	**7**
–	Body stout, moderate or relatively slender, depth at gill openings 2.4–4.2% TL	**8**
7	Body black to dark brown; teeth on maxilla uniserial at least in anterior part; total vertebrae 207–221	***O.macrochir* Bleeker, 1852**
–	Body pale brown; teeth on maxilla completely biserial; total vertebrae 178–184	***O.rotundus* Lee & Asano, 1997**
8	Dorsal-fin origin (DFO) equal or more than two pectoral-fin lengths behind gill openings	**9**
–	DFO in advance of, above, or behind gill openings by less than two (generally less than 1.5) pectoral-fin lengths	**15**
9	Eyes large, more than 70% of snout length	**10**
–	Eyes small to moderately large, less than 70% of snout length	**12**
10	Anal fin pale uniformly, without darkened base in advance of tail tip	***O.pratasensis* Ho, Ng & Lin, 2022**
–	Anal fin pale but with darkened base in advance of tail tip	**11**
11	SO 1+4, 3 preopercle pores; anterior tube mostly white; total vertebrae 157–168	***O.megalops* Asano, 1987**
–	SO 1+3, 2 preopercle pores; anterior tube brownish; total vertebrae 176	***O.semilunatus* Hibino & Chiu, 2019**
12	Body pale brown, bicolored; tail 53–57% TL	***O.bicolor* McCosker & Ho, 2015**
–	Body darker, uniformly dark brown or weakly pale on abdomen but with melanophores; tail 58–62% TL	**13**
13	Teeth numerous, vomer maximum 4 rows, maxilla to 4 or 5 rows; 5 or 6 mandibular pores; median fins white	***O.multidentis* sp. nov.**
–	Teeth moderate, vomer and maxilla maximum 2 rows; 6 mandibular pores; median fins dusky to dark brown	**14**
14	DFO behind gill opening by less than two pectoral-fin lengths; HL 8.9–11% TL	***O.aphotistos* McCosker & Chen, 2000**
–	DFO behind gill opening by more than three pectoral-fin lengths; HL 7.3–9.1% TL	***O.kusanagi* Hibino, McCosker & Tashiro, 2019**
15	DFO above or slightly behind level of gill openings; body with obscure distorted bars (both in life and preservation) but sometime faded; sensory pit black, conspicuous	***O.zophistius* (Jordan & Snyder, 1901)**
–	DFO clearly behind level of gill openings; body without bars; sensory pit same as body color, not conspicuous	**16**
16	Body pale to moderate, abdomen with or without scattered melanophores	**17**
–	Body uniformly dark or abdomen paler but completely covered by melanophores	**22**
17	One low labial protrusion (or rarely absent); posterior nostril opening outside mouth	**18**
–	One or two thorn-shaped labial protrusions; posterior nostril opening inside mouth	**19**
18	DFO before pectoral-fin tips; tip of tail stout, skin strongly wrinkled; head and body pale yellowish brown when fresh	***O.asakusae* Jordan & Snyder, 1901**
–	DFO usually behind pectoral-fin tips; tip of tail rather slender, skin smooth; head and body yellowish or reddish brown when fresh	***O.urolophus* (Temminck & Schlegel, 1846)**
19	One labial protrusion; lateral-line pores before anus 69–72; tail 50–52% TL; 1 teeth row on vomer	***O.shaoi* McCosker & Ho, 2015**
–	Two labial protrusions; lateral-line pores before anus 51–59; tail 53–67% TL; 2 or more teeth rows on vomer	**20**
20	Preopercle pores with dark margin; dorsal fin with dark narrow margin; total vertebrae 138–141	***O.apicalis* (Anonymous, 1830)**
–	Preopercle pores not margined; dorsal fin without dark margin, except for the darker rear portion	**21**
21	Tail 53–61% TL; preanal vertebrae 52–59; snout rather sharp	***O.machidai* McCosker, Ide & Endo, 2012**
–	Tail 61–67% TL; preanal vertebrae 48–52; snout rather swollen	***O.sangjuensis* (Ji & Kim, 2011)**
22	Lower-jaw tip anterior to base of anterior nostril tube; 6 or 7 mandibular pores; tooth rows on maxilla with a short additional row posteriorly	***O.kbalanensis* sp. nov.**
–	Lower-jaw tip below about middle of base of anterior-nostril tube; 4 or 5 mandibular pores; tooth rows on maxilla without a short additional row posteriorly	***O.obtusus* McCosker, Ide & Endo, 2012**

## ﻿Discussion

### ﻿Diversity of the genus *Ophichthus* in Taiwan

There is confusion surrounding some records of *Ophichthus* species in Taiwan, probably due to the close similarity and presence of many unknown species. A total of 23 species were included in the key to species of Taiwan above, although we expect more species come out in the near future. The species composition largely overlaps with that of Japanese waters, except some rare species in each country.

Some changes were made among the previously recorded species. For example, *Ophichthusfasciatus* Ju, Wu & Jin, 1981 has been synonymized under *Ophichthuszophistius* (Jordan & Snyder, 1901) by Hibino and McCosker (2020). The records of the narrowly distributed species *Ophichthusaltipennis* (Kaup, 1856) from Taiwan (Ho et al. 2015; McCosker and Ho 2015) are reidentified as *O.zophistius* by Y.H. *Ophichthysstenopterus* Cope, 1871 was synonymized under *Ophichthuspallens* (Richardson, 1848) by Hibino et al. (2019b). *Ophichthuspallens* was originally described based on a specimen collected by John Reeves from China, but no precise locality was provided. No confirmed voucher of *O.pallens* can be found from Taiwan (incl. John E. McCosker pers. com.), and it is excluded here from the Taiwanese fauna.

*Ophichthusretrodorsalis* Liu, Tang & Zhang, 2010 was described from the Taiwan Strait. Although it was originally placed in *Ophichthus*, we exclude it from this work and tentatively include it in *Pisodonophis*, based on H.C.H.’s examination of the holotype and an additional specimen collected from Ke-tzu-liao (NMMB-P28996, 622 mm TL). Both specimens have all jaw and vomer teeth blunt, molariform or granular and are closely similar to *Pisodonophisboro*. Future studies may prove that both species are synonymous.

We keep *Ophichthusapicalis* (Anonymous, 1830) in the *Ophichthus* fauna of Taiwan. However, no voucher specimen has been recognized from Taiwanese waters (Hibino pers. obs.). The species was originally described from Sumatra, Indonesia, and recorded as widespread in the Indo-West Pacific. However, the true *O.apicalis* has not been well defined, and its taxonomic status is still uncertain (Hibino pers. data). We tentatively keep *O.apicalis* in the key, with the data taken from specimens collected from Vietnam and Thailand, South China Sea (see comparative materials).

Hibino (2019) reported newly collected specimens of *Ophichthusrotundus* Lee & Asano, 1997 and *Ophichthussangjuensis* (Ji & Kim, 2011) from Taiwanese waters. The former is based on an old specimen collected from southwestern Taiwan in 1965. Many specimens of *O.sangjuensis* were also found in the collection, and some of them were previously misidentified as *Ophichthusmachidai* McCosker, Ide & Endo, 2012. The vomerine tooth arrangement for *Ophichthusmachidai* was formerly defined as biserial centrally and uniserial anteriorly and posteriorly (McCosker et al. 2012); however, we found that there is some variability in this character, some individuals have biserial or triserial tooth bands on vomer.

Among the Taiwanese species, *O.urolophus* is the most common and abundant and was collected from continental shelf and upper continental slope to depths of about 400 m. Despite few species being common inhabitants in coral reef areas, *O.asakusae*, *Ophichthuserabo* (Jordan & Snyder, 1901), *O.machidai*, and *O.sangjuensis* are also commonly seen in the bycatches of bottom trawlers, especially in the shallow waters of the west coast of Taiwan. Conversely, *O.lithinus* (Jordan & Richardson, 1908) is quite common in the catches of small set nets or fyke nets set up in mouths of rivers of southwestern Taiwan. Other species are either uncommon, rare, or rarely seen in the bycatches of fish landing grounds.

### ﻿Comparative materials

*Ophichthusapicalis*: FRLM 49738, 412 mm TL, FRLM 51386, 425 mm TL, FRLM 51388, 434 mm TL, FRLM 51390, 502 mm TL, FRLM 51391, 477 mm TL, Ha Long Bay, Vietnam; NSMT-P 104757, 361–380 mm TL (2), fish market at Songkhla, Thailand. *Ophichthusmachidai*: KAUM–I. 113140, 363 mm TL, NMMB-P23329, 435 mm TL, NMMB-P23330, 414 mm TL, NMMB-P23333, 443 mm TL, NMMB-P23334, 407 mm TL, NMMB-P27925, 456 mm TL, NMMB-P28997, 504 mm TL, Ke-tzu-liao, Kaohsiung, Taiwan. *Ophichthusobtusus*: NMMB-P16467, 438 mm TL, NMMB-P21764, 387 mm TL, Dong-gang, Taiwan. *Ophichthusrotundus*: NMMB-P05312, 475 mm TL, Shao-liu-qiu, Taiwan. *Ophichthussangjuensis*: NMMB-P23331, 468 mm TL, NMMB-P23332, 473 mm TL, NMMB-P28998, 316 mm TL, Ke-tzu-liao, Kaohsiung, Taiwan. *Pisodonophisretrodorsalis*: ASIZB [now NZMC] 50929, 517 mm TL, Taiwan Strait, Fujian, China; NMMB-P28996, 622 mm TL, Ke-tzu-liao, Kaohsiung, Taiwan.

## Supplementary Material

XML Treatment for Ophichthus kbalanensis

XML Treatment for Ophichthus multidentis

## ﻿Additional information

### Author ORCIDs

Yusuke Hibino https://orcid.org/0000-0002-5670-3851

Hsuan-Ching Ho https://orcid.org/0000-0003-1154-601X

Jian-Fu Huang https://orcid.org/0000-0001-5213-886X

### Data availability

All of the data that support the findings of this study are available in the main text.
